# Insights into plant‐part‐specific N_2_O production in roots and shoots of chicory (*Cichorium intybus*) using stable isotope labelling

**DOI:** 10.1111/nph.71565

**Published:** 2026-09-15

**Authors:** Moritz Schroll, Maurice Maas, Steffen Greiner, Thomas Klintzsch, Frank Keppler

**Affiliations:** ^1^ Institute of Earth Sciences Heidelberg University Heidelberg 69120 Germany; ^2^ Centre for Organismal Studies (COS) Im Neuenheimer Feld 360 Heidelberg 69120 Germany; ^3^ Heidelberg Center for the Environment (HCE) Heidelberg University Heidelberg 69120 Germany

**Keywords:** ammonium, *Cichorium intybus*, nitrate, nitrous oxide, plants, root, shoot, stable isotope labelling

## Abstract

- Nitrous oxide (N_2_O) contributes substantially to climate change and stratospheric ozone degradation, yet large uncertainties in its global budget point to overlooked sources. Growing evidence suggests plants produce N_2_O, but the mechanisms remain poorly constrained.
- For the first time, we resolved organ‐specific N_2_O formation in roots and shoots of aseptically cultivated chicory (*Cichorium intybus*) under light and dark conditions, combining ^15^N‐labelling (^15^NO_3_^−^/^15^NH_4_^+^) with position‐specific isotope analysis to identify N_2_O formation pathways.
- All cultures produced N_2_O under dark conditions, whereas illuminated shoots showed reduced production or net uptake, indicating photosynthetically driven suppression or internal consumption. Roots supplied with ^15^NO_3_^−^ exhibited relatively symmetric ^15^N enrichment at the α‐ and β‐positions of emitted N_2_O (compared to shoots), consistent with a reductive pathway that may involve nitric oxide as an intermediate. By contrast, shoots showed asymmetric intramolecular ^15^N enrichment exclusively under dark conditions with ^15^NO_3_^−^, indicating a NO_3_^−^‐dependent pathway mechanistically distinct from that operating in roots. No significant ^15^N incorporation was detected in any other shoot treatment.
- These organ‐specific, light‐dependent dynamics are absent from global N_2_O budgets and vegetation models. Our findings reveal unrecognized complexity in plant N_2_O exchange and provide a mechanistic framework for incorporating vegetation processes into future N_2_O budget assessments.

## Graphical abstract

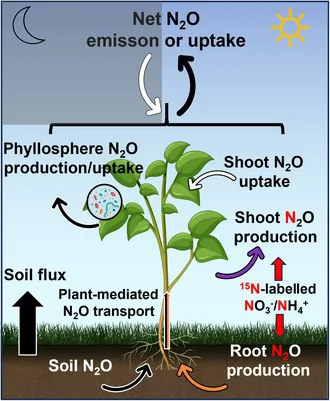

## Introduction

Nitrous oxide (N_2_O) is the third most important anthropogenic greenhouse gas, contributing to climate forcing and likely emerging as the dominant ozone‐depleting substance emitted during the 21^st^ century (Forster *et al*., 2007; Ravishankara *et al*., 2009). Its atmospheric concentration has increased by *c*. 22% compared to preindustrial levels, underscoring its growing impact on global climate and stratospheric ozone depletion (IPCC, 2014; Tian *et al*., 2020). Nitrogen, a vital macronutrient, plays a crucial role in plant growth and development. It is a fundamental component of Chl, amino acids, nucleic acids, and other secondary metabolites. Plants primarily acquire nitrogen in the forms of nitrate (NO_3_^−^) and ammonium (NH_4_^+^), which become available in the soil through natural processes such as nitrification and ammonification, as well as through fertilizers (Zayed *et al*., 2023). The microbial conversion of NO_3_^−^ and NH_4_^+^ to N_2_O through nitrification, nitrifier‐denitrification, and denitrification is responsible for two‐thirds of N_2_O emissions to the atmosphere (Tian *et al*., 2020).

Traditionally, studies on N_2_O cycling in terrestrial ecosystems have focussed on the exchange of N_2_O between soil surfaces and the atmosphere. While plants have primarily been recognized as conduits for N_2_O transfer in the soil–plant–atmosphere system, their own specific role in N_2_O formation and involved processes have surprisingly only recently been more in the focus of scientific interest. Already in the 1980s, Dean & Harper (1986) described the formation of N_2_O from two different plants species. Since then, an increasing number of studies observed N_2_O production and subsequent emission from vegetation including trees, plants, shrubs, herbs, cryptogamic covers, and grass (Dean & Harper, 1986; Chen *et al*., 1999; Goshima *et al*., 1999; Smart & Bloom, 2001; Hakata *et al*., 2003; Pihlatie *et al*., 2005; Bruhn *et al*., 2014; Lenhart *et al*., 2015, 2019; Machacova *et al*., 2016). However, there are contrasting findings, with several studies reporting N_2_O formation from the plants themselves, for example, when grown under sterile conditions (e.g. Lenhart *et al*., 2019) opposed to N_2_O production in plants attributed mainly to endophytic and phyllospheric bacteria (Bowatte *et al*., 2015; Qin *et al*., 2024). This contrasting evidence clearly shows that, even almost 40 yr after its first description, there is a clear lack of understanding of the underlying processes linked with plant N_2_O formation. This is further highlighted by the reported N_2_O emission rates from plants showing a large variability ranging from 10 to 6000 pmol g^−1^ h^−1^ (Lenhart *et al*., 2019; Qin *et al*., 2024). Nevertheless, recent studies by Lenhart *et al*. (2019) and Qin *et al*. (2024) estimate that plants contribute between 6% and 11% of global annual N_2_O emissions. These findings suggest that plant‐mediated N_2_O emissions are a ubiquitous process and highlight their potentially substantial role in the global N_2_O cycle. Despite this, the mechanisms of N_2_O formation in plants are mostly unclear and terrestrial vegetation has not yet been considered in global N_2_O budgets (Lenhart *et al*., 2019).

Previous studies have started to provide insight into the N_2_O formation mechanism using stable isotope techniques, since it allows for the differentiation of multiple sources due to their individual isotopic fingerprint (Toyoda *et al*., 2017). Position‐specific isotope analysis of N_2_O distinguishes the isotopic composition at the central (α) and terminal (β) nitrogen positions within the molecule. Because the two nitrogen atoms in N_2_O can originate from different precursors or reaction steps depending on the formation pathway, the position‐specific isotopic composition provides a powerful tool for mechanistic pathway attribution without requiring additional biochemical assays. Smart & Bloom (2001), by employing ^15^N‐labelled NO_3_^−^ as a tracer, demonstrated that enzymatic processes in plant foliage can generate N_2_O. Hakata *et al*. (2003) observed the emission of ^15^N‐labelled N_2_O from 16 different plant taxa grown aseptically in a ^15^N‐labelled NO_3_^−^ medium, concluding that NO_3_^−^ conversion to N_2_O is widespread in plants. Lenhart *et al*. (2019) were the first to report isotopic signatures of N_2_O released by plants, in addition to measuring the emission rates of 32 plant species from 22 families under controlled laboratory conditions. Emerging research suggests that plants possess the ability to denitrify (Timilsina *et al*., 2020, 2022a). For instance, mitochondria may serve as sites for N_2_O formation in plant cells, with evidence showing that plant N_2_O fluxes correlate positively with respiration rates and negatively with surface areas (Timilsina *et al*., 2022a). These relationships suggest that roots and mitochondria are integral to N_2_O production in natural conditions. Mechanistically, NO_3_^−^ serves as the primary precursor for plant‐derived N_2_O, being reduced via nitrate reductase to NO, which can subsequently be converted to N_2_O either via mitochondrial reduction under low‐O_2_ conditions or through the phytoglobin–NO cycle. The latter additionally serves to mitigate nitro‐oxidative stress under hypoxia (Timilsina *et al*., 2020).

Building on existing laboratory studies clearly identifying N_2_O formation from plants, two recent studies addressed the measurement of plant‐related N_2_O formation in the field and disentangling this production pathway from soil N_2_O fluxes using the stable isotope composition of N_2_O (Timilsina *et al*., 2022b; Xia *et al*., 2024). By comparing the isotopic signatures and emissions of N_2_O released from plants and soils, these studies unambiguously showed that plants are not merely passive conduits for N_2_O produced in soils but are themselves active sources contributing to atmospheric N_2_O fluxes. Moreover, there is further emerging evidence that N_2_O is not only produced by plants but that plants are also capable of reducing N_2_O fluxes, especially under photosynthetic activity (Schützenmeister *et al*., 2020). This finding has led to the hypothesis that N_2_O formation in plants may be more locally linked with the root systems (Timilsina *et al*., 2022b). However, currently, no study has systematically investigated the difference between N_2_O formation in different organs of plants such as the root and shoot.

To address this knowledge gap, we designed an isotope‐labelling study using chicory (*Cichorium intybus*) root and shoot cultures, cultivated under sterile conditions and guided by three specific hypotheses. First, we hypothesize that both roots and shoots are capable of producing N_2_O under conditions that minimize microbial contributions but that roots exhibit higher N_2_O fluxes per unit biomass than shoots owing to their higher respiratory activity and potentially lower internal oxygen availability. Second, we hypothesized that roots and shoots form N_2_O through mechanistically distinct pathways, reflected in different ^15^N enrichment patterns at the α‐ and β‐nitrogen positions of produced N_2_O. Third, building on previous evidence that photosynthetic activity reduces plant N_2_O emissions (Schützenmeister *et al*., 2020; Machacova *et al*., 2024), we hypothesized that light alters N_2_O exchange at the shoot organ level under sterile conditions.

## Materials and Methods

### Cultivation of root and shoot cultures of *Cichorium intybus*

Chicory (*Cichorium intybus* L.) was selected as the model organism for this study because its biology permits the independent cultivation of morphologically intact root and shoot cultures under fully sterile conditions (Kusch *et al*., 2009), enabling the attribution of N_2_O production to a single‐plant organ without the confounding influence of the other plant part or of associated soil microorganisms as well as considerable in‐house practical experience in the sterile cultivation. Chicory root cultures were established and cultivated following the methodology described by Kusch *et al*. (2009), resulting in hairy root cultures (HRCs) that were most comparable to young, fine roots. Root cultures were maintained in Erlenmeyer flasks filled with liquid medium and cultivated at a temperature of 25°C in a shaking incubator at 90 rpm in darkness. For chicory shoot cultures, chicory seeds were first treated with sodium hypochlorite and 0.01% Triton X‐100, followed by submersion in ethanol and subsequent rinsing with sterile water. Seeds were then germinated on Gamborg B5 medium (Duchefa Biochemie, Haarlem, the Netherlands) supplemented with 1% sucrose and 0.8% plant agar (Duchefa Biochemie) with a light phase (16 h) and a dark phase (8 h). Six weeks after germination, the seedlings were cut and the shoot segments were separately cultivated.

### Experimental set‐up and conditions

The experimental set‐up of this study consisted of separately incubated chicory shoots and roots with the addition of ^15^N‐labelled NH_4_^+^ and NO_3_^−^ solutions. Stable isotope labelled solutions were prepared by dissolving ^15^N‐labelled ammonium chloride (NH_4_Cl, ^15^N ≥ 98%, Sigma‐Aldrich) and sodium nitrate (NaNO_3_, ^15^N ≥ 98%, Sigma‐Aldrich) in demineralized and autoclaved water. The final concentrations of NH_4_Cl and NaNO_3_ ranged between 27 and 34 mmol l^−1^. All incubated roots and shoot cultures as well as the respective controls were incubated in airtight 580 ml glass flasks (Weck, Öflingen, Germany) equipped with silicone stoppers (Saint‐Gobain Performance Plastics, Charny, France) for headspace sampling. Root cultures were incubated in liquid Gamborg B5 medium at 23°C and darkness. Accordingly, root controls consisted of the same liquid medium and glass flasks that were used for root cultures, but without the presence of the roots. Chicory shoots were incubated with a Gamborg B5 medium under both dark and light conditions to assess potential effects of light on N_2_O formation and isotopic composition. Control experiments consisted of the same medium without shoot cultures. A volume of 10 ml of the prepared ^15^N‐labelled solutions was added to the shoot and root cultures as well as controls using a laminar flow bench. Additionally, shoots and controls were incubated in the dark without the addition of ^15^N–NH_4_^+^ or ^15^N–NO_3_^−^ solutions (Table 1). Due to technical difficulties, no root and ‘shoot + light’ without added labelled compounds could be investigated. Following the addition of the labelled solutions, the glass flasks were transferred to a climate chamber with a temperature of 25°C. Dark experiments were carried out with the chamber lights switched off, whereas light conditions followed a day‐night cycle with a 16 h light phase and an 8 h dark phase. The intensity of photosynthetically active radiation was *c*. 90 μmol s^−1^ m^−2^.

**Table 1:** Overview of incubation experiment set‐up.

Plant part	Treatment	Light condition	*n*
Root culture	^15^N–NO_3_^−^	Dark	3/4
	^15^N–NH_4_^+^		
Control	^15^N–NO_3_^−^	Dark	2
	^15^N–NH_4_^+^		
Shoot culture	^15^N–NO_3_^−^	Light	2/4
	^15^N–NH_4_^+^		3/4
	^15^N–NO_3_^−^	Dark	3/4
	^15^N–NH_4_^+^		4
	None		3/4
Control	^15^N–NO_3_^−^	Light	3
	^15^N–NH_4_^+^		
	^15^N–NO_3_^−^	Dark	4
	^15^N–NH_4_^+^		
	None		

For each treatment, the table lists the incubated *Cichorium intybus* plant part, the ^15^N‐labelled nitrogen source added, either labelled nitrate (^15^NO_3_^−^) or ammonium (^15^NH_4_^+^), the light condition, and the number of independent replicates (*n*).

### Gas sampling

First, the internal pressure inside the glass flasks was measured using a handheld manometer (GHM Messtechnik GmbH, Erolzheim, Germany) to allow the calculation of the exact amount of substance of target gases. Subsequently, for the analysis of N_2_O mixing ratios and its isotopic composition, a plastic syringe (Plastipak, Plymouth, MI, USA) was used to extract 60 ml of sample gas, which was transferred into 20 ml or 40 ml glass vials equipped with silicone–polytetrafluoroethylene (PTFE) septa or butyl–PTFE septa. For the analysis of oxygen (O_2_) and carbon dioxide (CO_2_) mixing ratios, a 7 ml gas sample was injected into 3 ml exetainers (Labco Ltd, London, UK). To compensate for the changing pressure inside the glass flasks resulting from gas sampling, a cannula equipped with a 0.2 μm filter (Cytiva, Marlborough, MA, USA) was used. Gas samples were collected at three different time points: 6, 24, and 48 h after start of the incubation. In addition, four laboratory air samples were taken at the start of the experiment to determine the initial gas composition within the glass flasks.

### Isotopes of N_2_O and definition of *δ*‐values

The four most common isotopologues of N_2_O are ^14^N^14^N^16^O, ^14^N^14^N^18^O, ^14^N^15^N^16^O, and ^15^N^14^N^16^O. The isotopomers ^14^N^15^N^16^O and ^15^N^14^N^16^O differ only in the position of the ^15^N atom within the molecule. When the ^15^N atom is located at the central nitrogen position, this configuration is referred to as ^15^N^α^, whereas its placement at the terminal position is defined as ^15^N^β^. The mean of the isotopic ratios of ^15^N^α^ and ^15^N^β^ is known as ^15^N^bulk^–N_2_O (Toyoda & Yoshida, 1999). In this study, all stable nitrogen isotope ratios are expressed using the conventional ‘delta’ (δ) notation (Eqn 1), referring to the relative difference of the isotope ratio of a sample from the *δ* ^15^ $N_{{\text{air−N}}_{2}}$ standard:

(Eqn 1) δN15=N15N14sampleN15N14air−N2−1

### Analysis of N_2_O mixing ratios and δ^15^N–N_2_O values

Analyses of N_2_O mixing ratios and its stable isotopes were performed using a Picarro G5131‐*i* cavity ring‐down spectroscope (CRDS; Picarro, Santa Clara, CA, USA). A Small Sample Introduction Module 2 (SSIM2; Picarro) was used to analyse small sample volumes ranging from 20 to 50 ml. Synthetic air (20.5% O_2_ in N_2_, ALPHAGAZ™ 1; Air Liquide, Paris, France) was used as carrier gas. Between each run, the system was evacuated and flushed with synthetic air to maintain accuracy. To ensure high‐quality analysis, two standard gases (RM2, δ^15^N^bulk^–N_2_O: +7.3‰, δ^15^N^α^–N_2_O: +15.8‰, δ^15^N^β^–N_2_O: −1.2‰; PIC14/2, δ^15^N^bulk^–N_2_O: +4.5‰, δ^15^N^α^–N_2_O: +15.9‰, δ^15^N^β^–N_2_O: −6.8‰) with known mixing ratios and isotopic composition were measured at the beginning and end of each measurement day for calibration and drift correction. CRDS instruments are known to be affected by cross‐sensitivities, where the occurrence and concentration of other gases can substantially influence the results of the analysis of the target gas (e.g. Harris *et al*., 2020). To prevent cross‐sensitivities with volatile organic compounds, a glass tube filled with Tenax© (Sigma‐Aldrich, USA) was placed in front of the inlet valve of the SSIM2 for the adsorption of volatile organic compounds. Additionally, the plastic syringe used for gas injection was partially filled the CO_2_ adsorbent Ascarite(II)© (Acros Organics, Geel, Belgium). Physical CO_2_ removal via Ascarite(II)© was applied to injected samples but was incomplete at high CO_2_ concentrations; for calibration standards introduced directly from tanks via the SSIM2, physical removal was not possible, so that in both cases, residual CO_2_ was additionally accounted for by spectroscopic correction. Corrections were applied for cross‐sensitivities to O_2_, CO_2_, CH_4_, and N_2_O concentration, as well as for effects of storage time, glass vials, and septa, following a similar approach of Havsteen *et al*. (2026), who developed a dedicated correction method for instruments of the same type (Supporting Information Methods S1–S3; Figs S1–S9). Although the applied corrections are non‐negligible, the underlying effects are systematic and could therefore be quantified and corrected. The accuracy of the corrected measurements was evaluated using the reference gases. The agreement between the corrected and assigned isotope values of the reference gases indicated that no substantial systematic bias remained after correction. The concentration ranges of interferants during the experiments and the magnitude of the corrections applied are described in the Methods S3. Analytical precision was assessed from repeated measurements of the working standard RM2 (Mohn *et al*., 2022) and ambient laboratory air, yielding 1*σ* values for RM2 of 0.5‰ for δ^15^N^bulk^–N_2_O, 0.4‰ for δ^15^N^α^–N_2_O, 0.8‰ for δ^15^N^β^–N_2_O, and 0.9‰ for δ^15^N^SP^–N_2_O, and for laboratory air of 0.4‰, 0.5‰, 0.4‰, and 0.6‰, respectively. Corresponding 3*σ* detection limits for RM2 were 1.4‰, 1.2‰, 2.4‰, and 2.7‰, and for laboratory air 1.2‰, 1.4‰, 1.2‰, and 1.8‰, respectively.

### Analysis of CO_2_ and O_2_ mixing ratios

CO_2_ and O_2_ mixing ratios were determined using a GC‐2010 Plus gas chromatograph (Shimadzu, Kyoto, Japan) coupled with a barrier discharge ionization detector (BID‐2010 Plus; Shimadzu). The set‐up included a packed stainless steel column with a length of 2 m, an inner diameter of 0.53 mm, and a carbon molecular sieve (ShinCarbon ST, 80/100 Mesh; Supelco, Bellefonte, PA, USA) as the stationary phase. High‐purity helium (grade 6.0; Air Liquide) was used as the carrier gas at a constant flow rate of 5 ml min^−1^. Sample injection was performed using an autosampler (AOC‐20i, Shimadzu). As part of quality control, one reference standard was measured after every six to nine single measurements. Calibration was conducted using certified standard gas mixtures (CRYSTAL Gas Mixture; Air Liquide). The quantification of CO_2_ and O_2_ was determined by analysing multiple reference standards (CO_2_: 400 ppm–40% by volume, O_2_: 1–22% by volume), with each measurement performed in triplicate.

### Mass balance approach for determination of isotopic composition of N_2_O

To characterize the isotopic composition of the N_2_O produced during each incubation, we applied a two‐component isotope mass balance approach. At time point *t2*, the total N_2_O in the flask headspace was treated as a mixture of the N_2_O present at the start of the incubation (*t*0) and the N_2_O produced by the plant tissue between *t*0 and *t2*. The isotopic composition of the produced N_2_O was calculated according to Eqn (2):

(Eqn 2) δNproduced15=xit2xVHSxδNt215−xit0xVHSxδNt015xit2−xit0

where *xi* represents the mixing ratio of N_2_O and *V*_HS_ represents the headspace volume at the respective time point. This calculation was applied identically to δ^15^N^bulk^–N_2_O, δ^15^N^α^, and δ^15^N^β^, yielding the position‐specific isotopic composition of produced N_2_O for each treatment. Values are reported with their associated SD from replicate measurements.

### Statistical methods

The evaluation of measurement data from the Picarro G5131‐*i* was performed using R and Microsoft Excel. For the analysis of raw data from the Picarro G5131‐*i*, moving averages of the measurements over 100 s were calculated. For each measurement, a stable measurement period of at least 5 min was selected. Mixing ratios, production rates, and the isotopic change rate are presented as the arithmetic mean (*n* = 3 or 4) of independent replicates with their SDs. For some samples, results at specific time points were discarded due to analytical problems and instrument failure, including sample loss within the Picarro system and carry‐over effects from previous measurements. If fewer than three measurements were available for a given time point, no calculation of the SD was carried out. Additionally, the obtained data were used to determine the production rates of the gases N_2_O, O_2_, and CO_2_ for the shoot and root cultures as well as for the controls. For each incubated glass flask, an individual production rate was determined using linear regression. The arithmetic means of these production rates were then calculated for the same plant compartment and treatment. Furthermore, *t*‐tests were performed using sigmaplot (v.12.2.0.45, USA) to statistically support the observed findings. Please note that this study follows the recommendations of the American Statistical Association, ensuring that *P*‐values and other statistical parameters were not used as the sole basis for drawing conclusions (Wasserstein *et al*., 2019). Consequently, the term ‘statistically significant’ was deliberately avoided in the interpretation of the results.

## Results

The incubations comprised a series of experiments in which shoot and root cultures of chicory were incubated under dark and light conditions with the addition of ^15^N‐labelled NO_3_^−^ and NH_4_^+^ to assess the changes in the mixing ratios of N_2_O, CO_2_, and O_2_ as well as the isotopic composition of N_2_O. The results in the first three subsections (Figs 1, 2, 3) present changes relative to the measurements at the start of the incubation. Furthermore, measured values are reported as absolute changes in the respective glass flasks, without considering the biomass of the different plant parts to ensure the comparability of flasks with plants and the respective controls, as the controls do not possess biomass. For the calculation of production rates, dry plant biomass was considered (Fig. 4). The isotopic composition of the N_2_O produced during each incubation was calculated via the two‐component isotope mass balance (Eqn 2; Fig. 5).

**Fig. 1:**
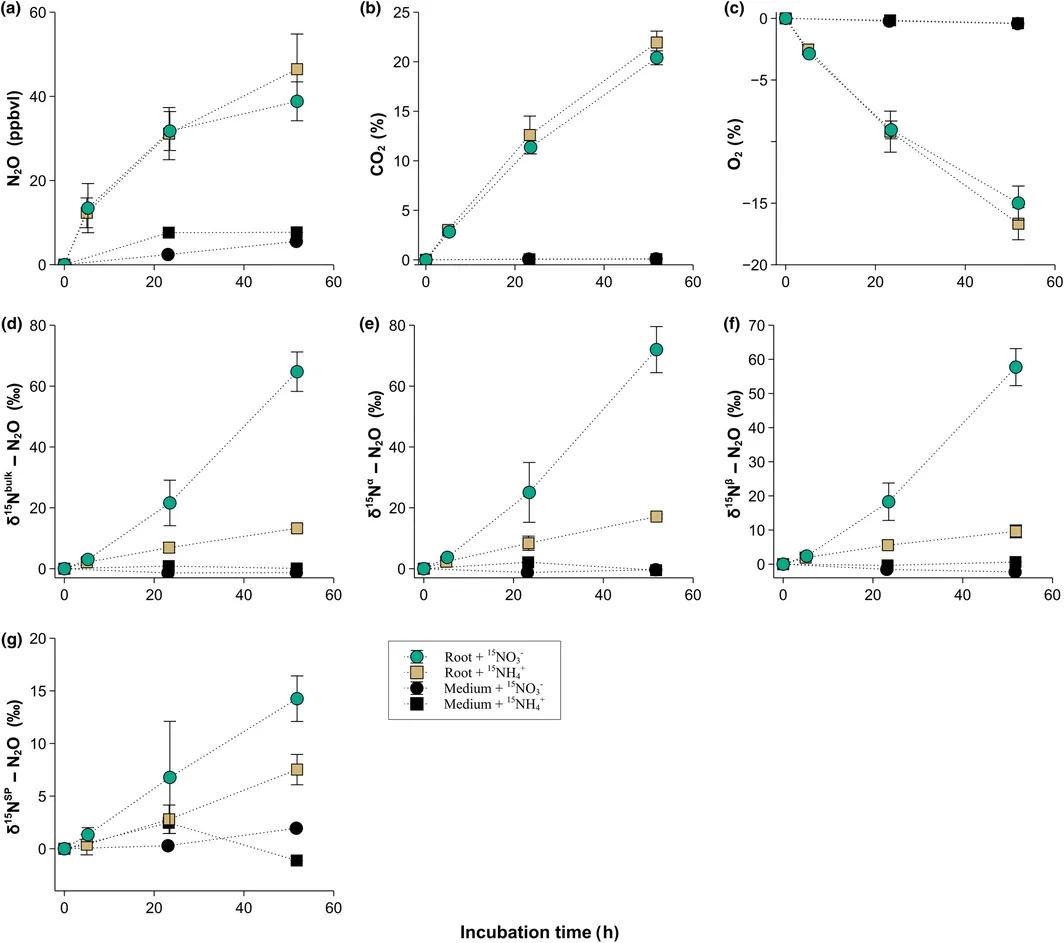
Time course of gas mixing ratios and of the bulk and intramolecular ^15^N isotopic composition of nitrous oxide (N_2_O) during dark incubation of *Cichorium intybus* root cultures following supplementation with ^15^NO_3_^−^ and ^15^NH_4_^+^. Panels show the relative changes over the incubation in the mixing ratios of (a) N_2_O, (b) carbon dioxide (CO_2_), and (c) oxygen (O_2_), and in (d) the bulk δ^15^N value of N_2_O (δ^15^N^bulk^), (e) the ^15^N value at the central α position (δ^15^N^α^), (f) the ^15^N value at the terminal β position (δ^15^N^β^), and (g) the ^15^N site preference (δ^15^N^SP^). Please note that the results are presented as changes relative to the measurements at the incubation start. Error bars represent the SD (*n* = 3 or 4). Reported values reflect absolute changes in the respective glass flasks and were not normalized to biomass or its variation over time.

### Chicory roots under dark conditions

N_2_O mixing ratios during the incubation of chicory roots increased by 38.8 ± 4.6 parts per billion by volume (ppbv) when supplemented with ^15^NO_3_^−^ (*n* = 3; Fig. 1a). The increase in N_2_O mixing ratios was accompanied by a strong shift in stable isotope values towards more positive values with δ^15^N^bulk^–N_2_O values increasing by +64.7 ± 6.5‰ (*n* = 3), δ^15^N^α^–N_2_O by +72.0 ± 7.6‰, δ^15^N^β^–N_2_O by +57.7 ± 5.4‰, and δ^15^N^SP^–N_2_O values by +14.3 ± 2.2‰ (*n* = 3; Fig. 1d–g). CO_2_ and O_2_ mixing ratios exhibited opposite trends, with CO_2_ increasing on average by 20.4 ± 0.7% (*n* = 3) while O_2_ mixing ratios decreased by 15.0 ± 1.1% (*n* = 3) (Fig. 1b,c).

When chicory roots were supplemented with ^15^NH_4_^+^, N_2_O mixing ratios increased by 46.5 ± 8.4 ppbv (*n* = 3; Fig. 1a). Compared to the treatment with NO_3_^−^, ‘root + ^15^NH_4_^+^’ showed a less pronounced shift towards more positive stable isotope values with δ^15^N^bulk^–N_2_O values increasing by +13.3 ± 1.3‰ (*n* = 3), δ^15^N^α^–N_2_O by +17.1 ± 0.7‰, δ^15^N^β^–N_2_O by +9.6 ± 1.9‰, and δ^15^N^SP^–N_2_O values by +7.5 ± 1.4‰ (*n* = 3; Fig. 1d–g). The δ^15^N^SP^–N_2_O value of produced N_2_O reached 7.5 ± 1.4‰ at the end of the incubation period (Fig. 1g), lower than in the ^15^NO_3_^−^ treatment but still reflecting preferential ^15^N enrichment at the α‐position. During the incubation period, CO_2_ mixing ratios increased by 21.9 ± 1.2% (*n* = 3) while O_2_ mixing ratios decreased by 16.7 ± 1.0% (*n* = 3).

Controls containing ‘medium + ^15^NO_3_^−^’ and ‘medium + ^15^NH_4_^+^’ showed only minor increases in N_2_O mixing ratios (5.5 ppbv for ‘medium + ^15^NO_3_^−^’ (*n* = 2) and 4.5 ppbv for ‘medium + ^15^NH_4_^+^’ (*n* = 2), respectively) and negligible changes in stable isotope values as well as CO_2_ and O_2_ mixing ratios during the incubation.

### Chicory shoots under dark conditions

N_2_O mixing ratios increased by 9.8 ± 2.8 ppbv when chicory shoots were incubated in the dark with ^15^NO_3_^−^ (*n* = 3; Fig. 2a). This increase was accompanied by a pronounced shift towards more positive δ^15^N values: +10.1 ± 1.4‰ for δ^15^N^bulk^–N_2_O, +15.8 ± 1.1‰ for δ^15^N^α^–N_2_O, +4.6 ± 2.5‰ for δ^15^N^β^–N_2_O, and for 11.2 ± 2.7‰ for δ^15^N^SP^–N_2_O (*n* = 3; Fig. 2b–d,g). CO_2_ mixing ratios increased by 3.28 ± 0.77% while O_2_ decreased by 3.91 ± 0.59% (*n* = 3; Fig. 2e,f).

**Fig. 2:**
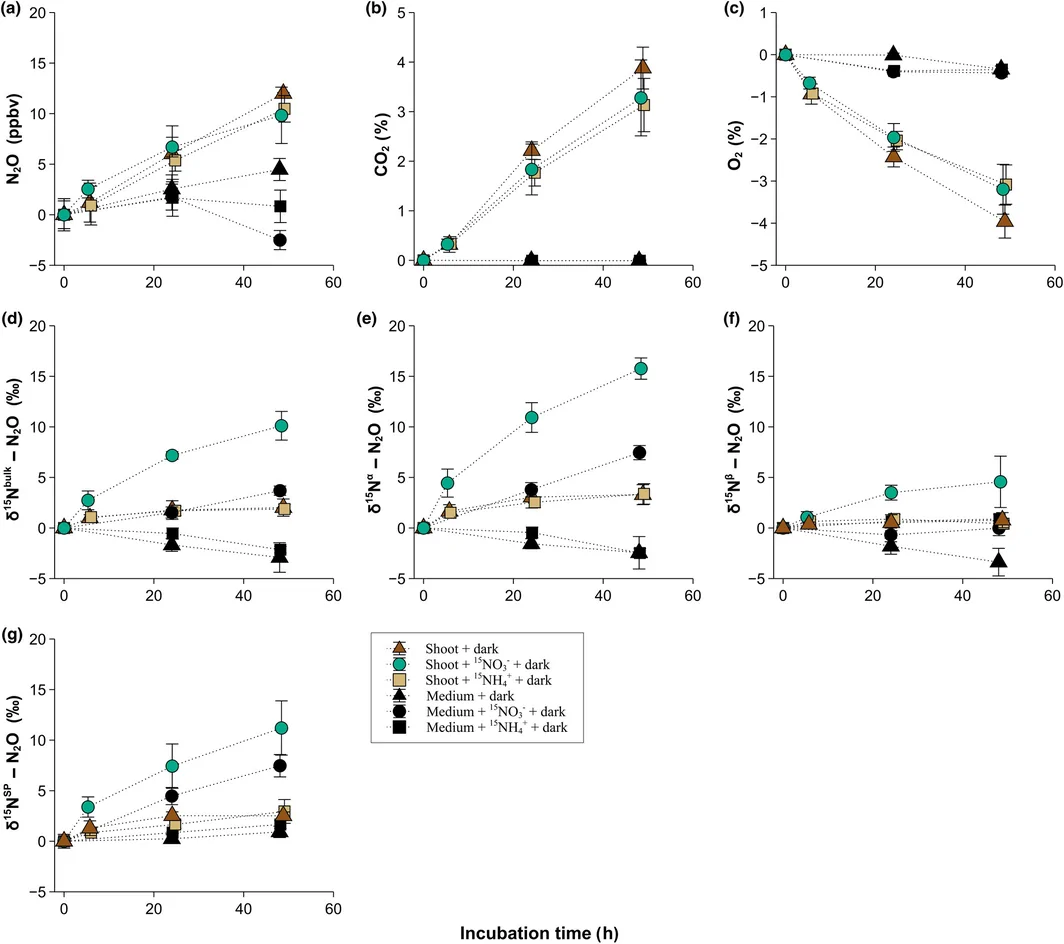
Time course of gas mixing ratios and of the bulk and intramolecular ^15^N isotopic composition of nitrous oxide (N_2_O) during dark incubation of *Cichorium intybus* shoots in dark conditions following supplementation with ^15^NO_3_^−^ and ^15^NH_4_^+^. Panels show the relative changes over the incubation in the mixing ratios of (a) N_2_O, (b) carbon dioxide (CO_2_), and (c) oxygen (O_2_), and in (d), the bulk δ^15^N value of N_2_O (δ^15^N^bulk^), (e) the ^15^N value at the central α position (δ^15^N^α^), (f) the ^15^N value at the terminal β position (δ^15^N^β^), and (g) the ^15^N site preference (δ^15^N^SP^). Please note that the results are presented as changes relative to the measurements at the incubation start. Error bars represent the SD (*n* = 3 or 4). Reported values reflect absolute changes in the respective glass flasks and were not normalized to biomass or its variation over time.

When shoots were incubated in the dark with ^15^NH_4_^+^, N_2_O mixing ratios elevated by 10.5 ± 1.3 ppbv (*n* = 4; Fig. 2a). Isotopic shifts were smaller accounting for +1.9 ± 0.5‰ for δ^15^N^bulk^–N_2_O, +3.4 ± 1.0‰ for δ^15^N^α^–N_2_O, +0.4 ± 0.3‰ for δ^15^N^β^–N_2_O, and 2.9 ± 1.2‰ for δ^15^N^SP^–N_2_O (*n* = 4; Fig. 2b–d,g). CO_2_ increased by 3.13 ± 0.54% and O_2_ decreased by 3.08 ± 0.47% (*n* = 4; Fig. 2e,f).

In the ‘shoot + dark only’ treatment, N_2_O mixing ratios rose by 12.0 ± 0.1 ppbv (*n* = 3), with δ^15^N^bulk^–N_2_O values increasing by +2.0 ± 0.9‰, δ^15^N^α^–N_2_O by +3.3 ± 1.0‰, δ^15^N^β^–N_2_O by +0.8 ± 0.7‰, and for δ^15^N^SP^–N_2_O by 2.5 ± 0.4‰ (*n* = 3; Fig. 2b–d,g). CO_2_ mixing ratios increased by 3.88 ± 0.42% and O_2_ mixing ratios decreased by 3.96 ± 0.39% (*n* = 3; Fig. 2e,f).

Control incubations (‘medium + dark + ^15^NO_3_^−^’; ‘medium + dark + ^15^NH_4_^+^’; ‘medium + dark only’) showed minimal N_2_O changes (−2.5 ± 1.0 ppbv, +0.8 ± 1.6 ppbv, and +4.5 ± 1.1 ppbv, respectively; *n* = 4) and negligible shifts in δ^15^N^bulk^–N_2_O, δ^15^N^α^–N_2_O, δ^15^N^β^–N_2_O, δ^15^N^SP^–N_2_O values, and CO_2_/O_2_ mixing ratios (< ±0.4%; *n* = 4).

### Chicory shoots under light conditions

The N_2_O mixing ratio increased by 15.4 ppbv when shoots were incubated in the light with ^15^NO_3_^−^ (*n* = 2; Fig. 3a). This was accompanied by a pronounced shift towards more positive δ^15^N values. δ^15^N^bulk^–N_2_O values increased by 14.4‰ (*n* = 2; Fig. 3d), δ^15^N^α^–N_2_O values by 14.1 ± 1.5‰, δ^15^N^β^–N_2_O values by 4.5 ± 2.0‰ (*n* = 3; Fig. 3e,f), and δ^15^N^SP^–N_2_O values by 9.6‰ (*n* = 2; Fig. 3g). Under the same conditions, CO_2_ mixing ratios increased by 4.20% and 4.33% (*n* = 2; Fig. 3b), respectively, while O_2_ decreased by 0.34% and 3.83% (*n* = 2; Fig. 3c).

**Fig. 3:**
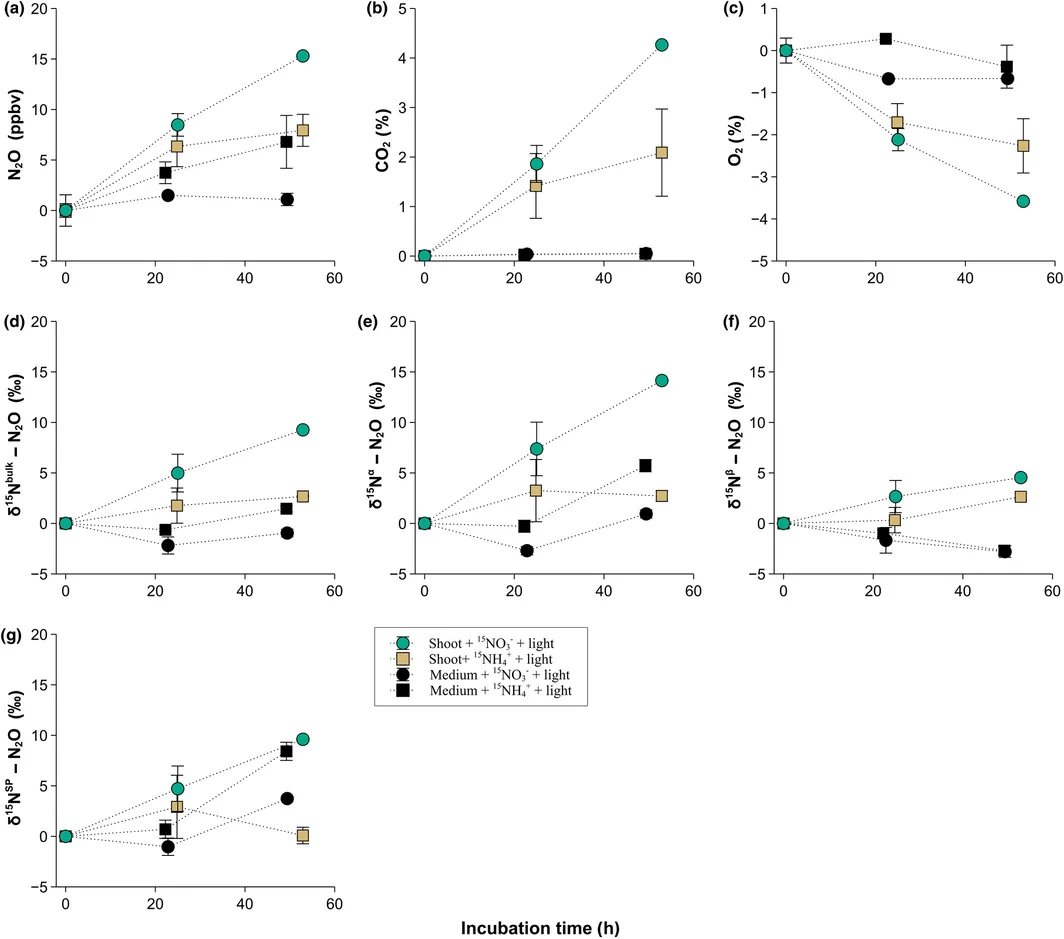
Time course of gas mixing ratios and of the bulk and intramolecular ^15^N isotopic composition of nitrous oxide (N_2_O) during incubation of *Cichorium intybus* shoots under light exposure following supplementation with ^15^NO_3_^−^ and ^15^NH_4_^+^. Panels show the relative changes over the incubation in the mixing ratios of (a) N_2_O, (b) carbon dioxide (CO_2_), and (c) oxygen (O_2_), and in (d), the bulk δ^15^N value of N_2_O (δ^15^N^bulk^), (e) the ^15^N value at the central α position (δ^15^N^α^), (f) the ^15^N value at the terminal β position (δ^15^N^β^), and (g) the ^15^N site preference (δ^15^N^SP^). Please note that the results are presented as changes relative to the measurements at the incubation start. Error bars represent the SD (*n* = 3 or 4). Reported values reflect absolute changes in the respective glass flasks and were not normalized to biomass or its variation over time.

When shoots were supplied with ^15^NH_4_^+^ under light exposure, the N_2_O mixing ratio increased by 7.9 ± 1.6 ppbv (*n* = 3; Fig. 3a). Shifts towards more positive δ^15^N^bulk^–N_2_O values were more modest (+2.7 ± 0.5‰; *n* = 3; Fig. 3d) and δ^15^N^α^–N_2_O increased by 2.7 ± 0.5‰, δ^15^N^β^–N_2_O by 2.6 ± 0.3‰, and δ^15^N^SP^–N_2_O values by 0.1 ± 0.8‰ (*n* = 3; Fig. 3e–g). CO_2_ mixing ratios increased by 2.09 ± 0.88% and O_2_ decreased by 2.26 ± 0.64% (*n* = 3; Fig. 3b,c).

Controls (‘medium + ^15^NO_3_^−^ + light’ and ‘medium + ^15^NH_4_^+^ + light’; *n* = 3) showed only minor increases in N_2_O mixing ratios of 1.1 ± 0.6 ppbv and 6.8 ± 2.6 ppbv, respectively (Fig. 3a). Isotopic shifts in these controls were negligible for δ^15^N^bulk^–N_2_O values (1.0 ± 0.4‰ and +1.5 ± 0.3‰), while δ^15^N^α^–N_2_O/δ^15^N^β^–N_2_O values changed by < 3‰ and δ^15^N^SP^–N_2_O values changed by +3.7 ± 0.2‰ in the ^15^NO_3_^−^ control (*n* = 2; Fig. 3d–f,g). CO_2_ and O_2_ mixing ratios remained within ±0.4% of initial values.

### Rates of N_2_O, CO_2_ production, and O_2_ uptake

Root cultures produced N_2_O with rates of 15.1 ± 4.1 pmol h^−1^ g^−1^ (*n* = 3) and 20.0 ± 5.1 pmol h^−1^ g^−1^ (*n* = 3, *P* = 0.89 when comparing these treatments) for NO_3_^−^ and NH_4_^+^ supplementation, respectively. Shoots incubated under dark conditions exhibited the highest N_2_O production rates when supplemented with either ^15^NO_3_^−^ (23.2 ± 3.7 pmol h^−1^ g^−1^, *n* = 3) or ^15^NH_4_^+^ (27.1 ± 6.0 pmol h^−1^ g^−1^, *n* = 4), while the ‘shoot + dark only’ treatment exhibited a production rate of 13.6 ± 2.8 pmol h^−1^ g^−1^ (*n* = 3, *P* < 0.05) (Fig. 4a). When shoots were incubated under light exposure, substantially lower N_2_O production rates were observed than those under dark conditions (*P* < 0.05). Specifically, the ‘shoot + ^15^NO_3_^−^ + light’ treatment yielded a production rate of 2.6 pmol h^−1^ g^−1^ (*n* = 2), while the ‘shoot + ^15^NH_4_^+^ + light’ treatment even showed a small net uptake of N_2_O, with a rate of −1.3 ± 0.8 pmol h^−1^ g^−1^ (*n* = 3).

**Fig. 4:**
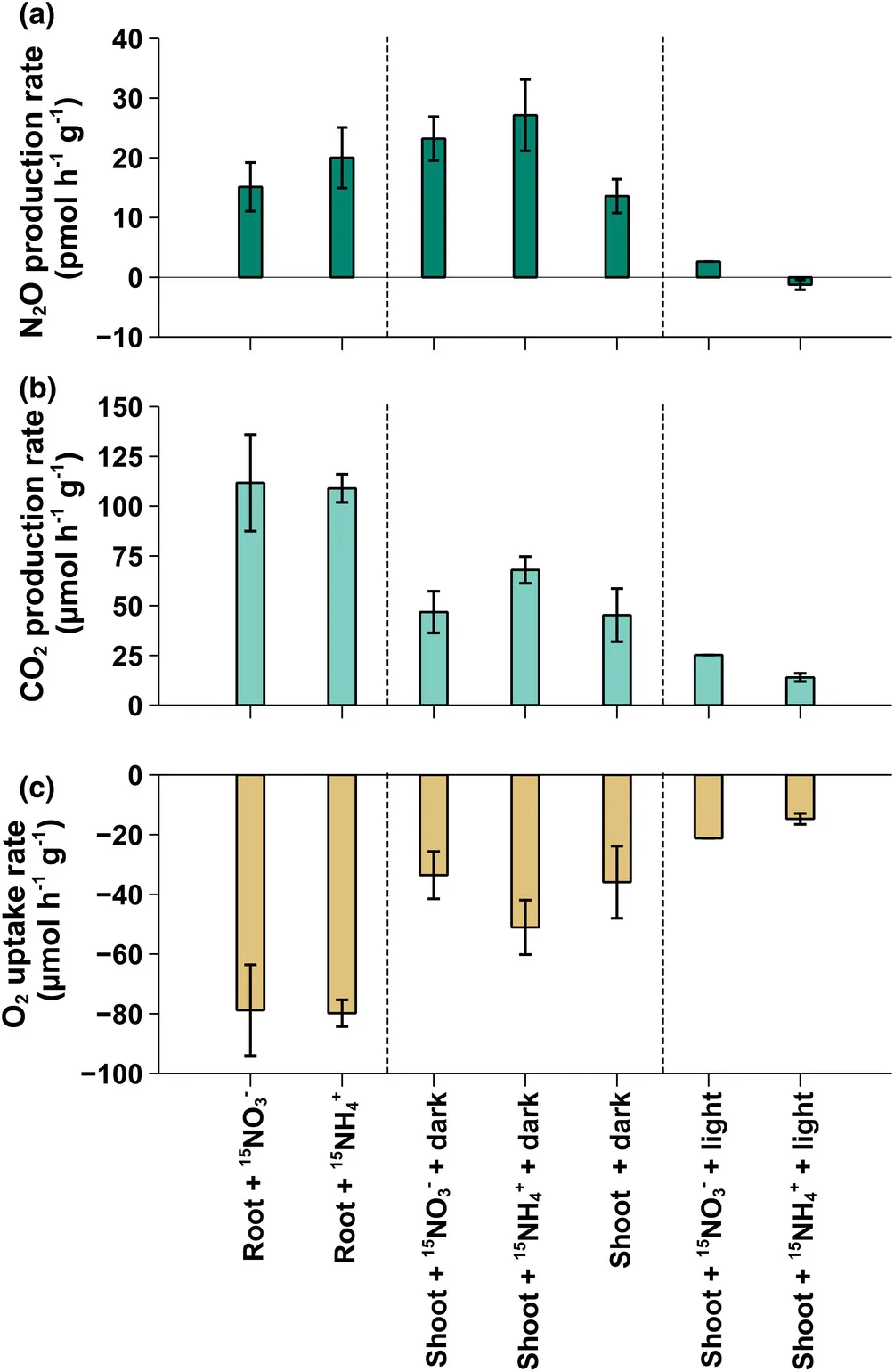
Production and uptake rates of *Cichorium intybus* root and shoot cultures across all incubation treatments, normalized to plant dry weight (pmol or μmol h^−1^ g^−1^). (a) Nitrous oxide production rates, (b) carbon dioxide production rates, and (c) oxygen uptake rates. Error bars reflect propagated SD (*n* = 3 or 4). Associated *P*‐values are shown in Supporting Information Fig. S10(a–c).

CO_2_ production rates were highest in ‘root + ^15^NO_3_^−^’ and ‘root + ^15^NH_4_^+^’ treatments, with rates of 111.7 ± 24.2 μmol h^−1^ g^−1^ (*n* = 3) and 109.0 ± 7.0 μmol h^−1^ g^−1^ (*n* = 3), respectively. Shoots incubated in the dark showed CO_2_ production rates of 46.8 ± 10.5 μmol h^−1^ g^−1^ (*n* = 3) for the ‘shoot + ^15^NO_3_^−^ + dark’ treatment, 68.0 ± 6.7 μmol h^−1^ g^−1^ (*n* = 4) for ‘shoot + ^15^NH_4_^+^ + dark’, and 45.3 ± 13.3 μmol h^−1^ g^−1^ (*n* = 3) for ‘shoot + dark only’. The lowest CO_2_ production rates were found in light‐incubated shoots treated with ^15^NO_3_^−^ (25.3 μmol h^−1^ g^−1^, *n* = 2) and ^15^NH_4_^+^ (14.0 ± 2.1 μmol h^−1^ g^−1^, *n* = 3).

Root cultures also exhibited the highest O_2_ consumption rates, with values of 78.8 ± 15.2 μmol h^−1^ g^−1^ (*n* = 3) and 79.8 ± 4.5 μmol h^−1^ g^−1^ (*n* = 3) for ^15^NO_3_^−^ and ^15^NH_4_^+^ treatments, respectively. Shoots incubated under dark conditions showed lower O_2_ uptake rates compared to roots, with rates of 33.6 ± 7.9 μmol h^−1^ g^−1^ (*n* = 3) for ‘shoot + ^15^NO_3_^−^ + dark’, 51.1 ± 9.1 μmol h^−1^ g^−1^ (*n* = 4) for ‘shoot + ^15^NH_4_^+^ + dark’, and 35.9 ± 12.1 μmol h^−1^ g^−1^ (*n* = 3) for the ‘shoot + dark only’ treatment. Light‐incubated shoots showed the lowest O_2_ consumption rates with 21.2 μmol h^−1^ g^−1^ (*n* = 2) when ^15^NO_3_^−^ and 14.7 ± 1.8 μmol h^−1^ g^−1^ (*n* = 3) when ^15^NH_4_^+^ was added.

### Isotopic composition of produced N_2_O

The isotopic composition of the N_2_O produced during each incubation, calculated via the two‐component isotope mass balance, is presented in Fig. 5. Values represent the δ^15^N^bulk^–N_2_O, δ^15^N^α^–N_2_O, δ^15^N^β^–N_2_O, and δ^15^N^SP^–N_2_O values of the N_2_O formed between the start and the end of the incubation, corrected for the ambient N_2_O present at the start of the incubation.

**Fig. 5:**
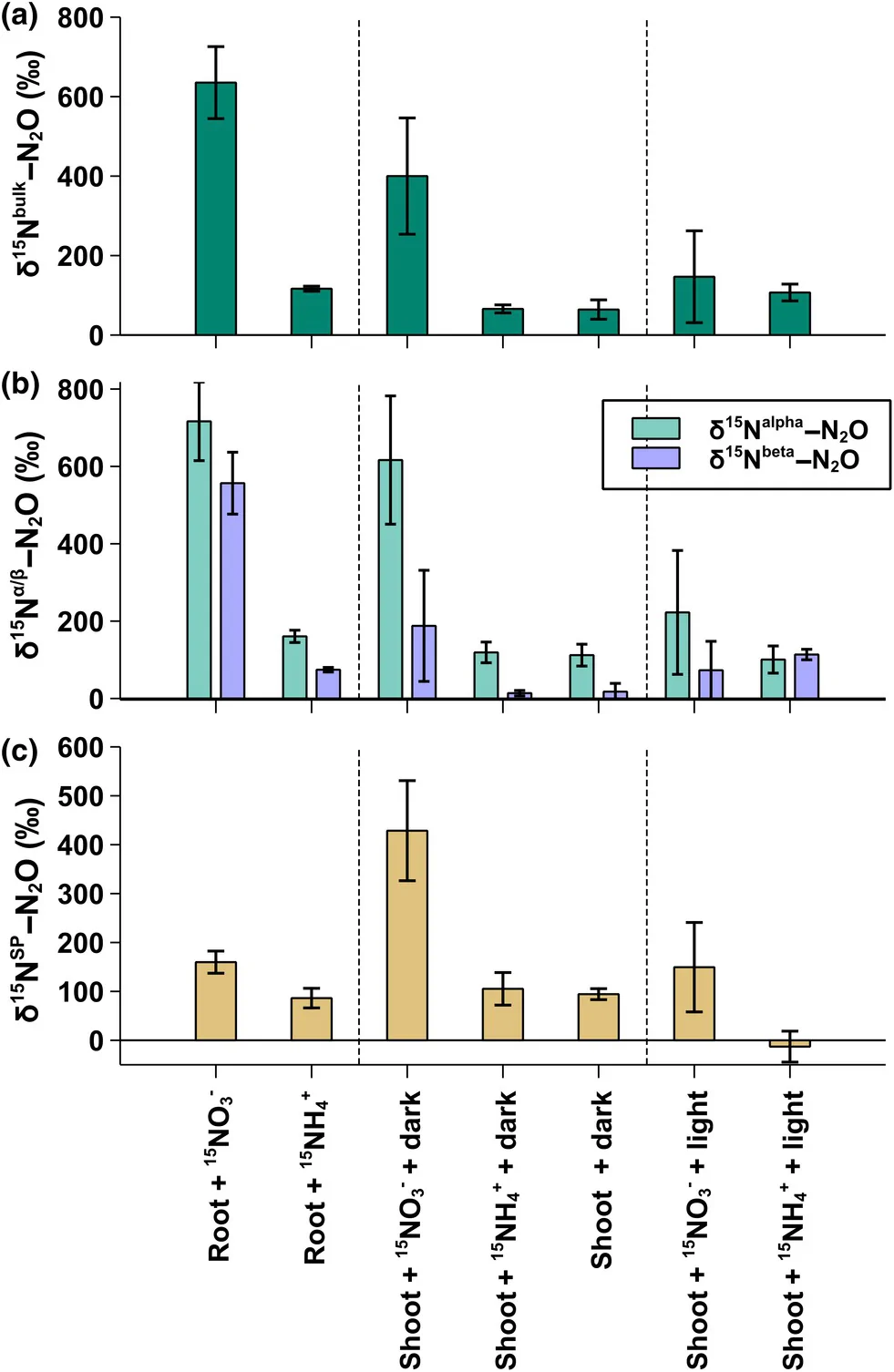
Isotopic composition of produced nitrous oxide (N_2_O) calculated via the two‐component isotope mass balance for each treatment and corrected for ambient N_2_O at the onset of incubation. (a) The bulk δ^15^N value of the produced N_2_O (δ^15^N^bulk^), (b) the δ^15^N values at the central α and terminal β positions (δ^15^N^α^ and δ^15^N^β^), and (c) the δ^15^N site preference (δ^15^N^SP^). Values represent the arithmetic mean of independent replicates; error bars denote one SD, except for the ‘shoot + ^15^NO_3_^−^ + light’ treatment (*n* = 2) where error bars denote the range between the two measurements. Associated *P*‐values are shown in Supporting Information Fig. S10(d–g).

Root cultures supplemented with ^15^NO_3_^−^ showed the highest isotopic values of produced N_2_O compared to all other root and shoot treatments (*P* < 0.02), with δ^15^N^bulk^–N_2_O values of 635 ± 91‰, δ^15^N^α^–N_2_O values of 716 ± 102‰, δ^15^N^β^–N_2_O values of 557 ± 80‰ and δ^15^N^SP^–N_2_O values of 160 ± 23‰ (*n* = 3; Fig. 5a–c). Root cultures supplemented with ^15^NH_4_^+^ showed substantially lower values across all isotopic parameters, with δ^15^N^bulk^–N_2_O values of 117 ± 6‰, δ^15^N^α^–N_2_O values of 161 ± 16‰, δ^15^N^β^–N_2_O values of 74 ± 6‰, and δ^15^N^SP^–N_2_O values of 86 ± 20‰ (*n* = 3; *P* = 0.01; Fig. 5a–c).

Among dark‐incubated shoot treatments, the ‘shoot + ^15^NO_3_^−^ + dark’ treatment showed the highest isotopic values with δ^15^N^bulk^–N_2_O values of 400 ± 146‰, δ^15^N^α^–N_2_O values of 616 ± 166‰, δ^15^N^β^–N_2_O values of 188 ± 144‰, and notably elevated δ^15^N^SP^–N_2_O values of 429 ± 102‰ (*n* = 3; Fig. 5a–c). By contrast, the ‘shoot + ^15^NH_4_^+^ + dark’ treatment showed considerably lower values with δ^15^N^bulk^–N_2_O values of 66 ± 10‰, δ^15^N^α^–N_2_O values of 119 ± 27‰, δ^15^N^β^–N_2_O values of 14 ± 7‰, and δ^15^N^SP^–N_2_O values of 105 ± 33‰ (*n* = 4; Fig. 5a–c), closely comparable to the ‘shoot + dark only’ treatment (δ^15^N^bulk^–N_2_O values: 64 ± 24‰, δ^15^N^α^–N_2_O values: 112 ± 28‰, δ^15^N^β^–N_2_O values: 18 ± 21‰, δ^15^N^SP^–N_2_O values: 94 ± 11‰; *n* = 3; Fig. 5a–c).

Under light conditions, the ‘shoot + ^15^NO_3_^−^ + light’ treatment showed δ^15^N^bulk^–N_2_O values of 147 ± 116‰, δ^15^N^α^–N_2_O values of 223 ± 160‰, δ^15^N^β^–N_2_O values of 73 ± 75‰ and δ^15^N^SP^–N_2_O values of 149 ± 91‰ (*n* = 2, ± range; Fig. 5a–c). The ‘shoot + ^15^NH_4_^+^ + light’ treatment showed δ^15^N^bulk^–N_2_O values of 107 ± 21‰, δ^15^N^α^–N_2_O values of 101 ± 35‰, δ^15^N^β^–N_2_O values of 114 ± 14‰ and δ^15^N^SP^–N_2_O values of −13 ± 32‰ (*n* = 3; Fig. 5a–c).

## Discussion

### Plant‐mediated N_2_O cycling as an organ‐specific process: a revised conceptual framework

Our results demonstrate for the first time at the plant organ level that root and shoot N_2_O cycling operates through distinct biochemical pathways responding differently to NO_3_^−^/NH_4_^+^ availability and light regime. This extends the current conceptual framework of plant roles in the soil–plant–atmosphere continuum: beyond soil‐derived N_2_O flux, plant‐mediated transport, and phyllosphere interactions, plants harbour an intrinsic, organ‐specific set of N_2_O production and consumption pathways that fundamentally alter how plant contributions to atmospheric N_2_O should be conceptualized and quantified (Fig. 6).

**Fig. 6:**
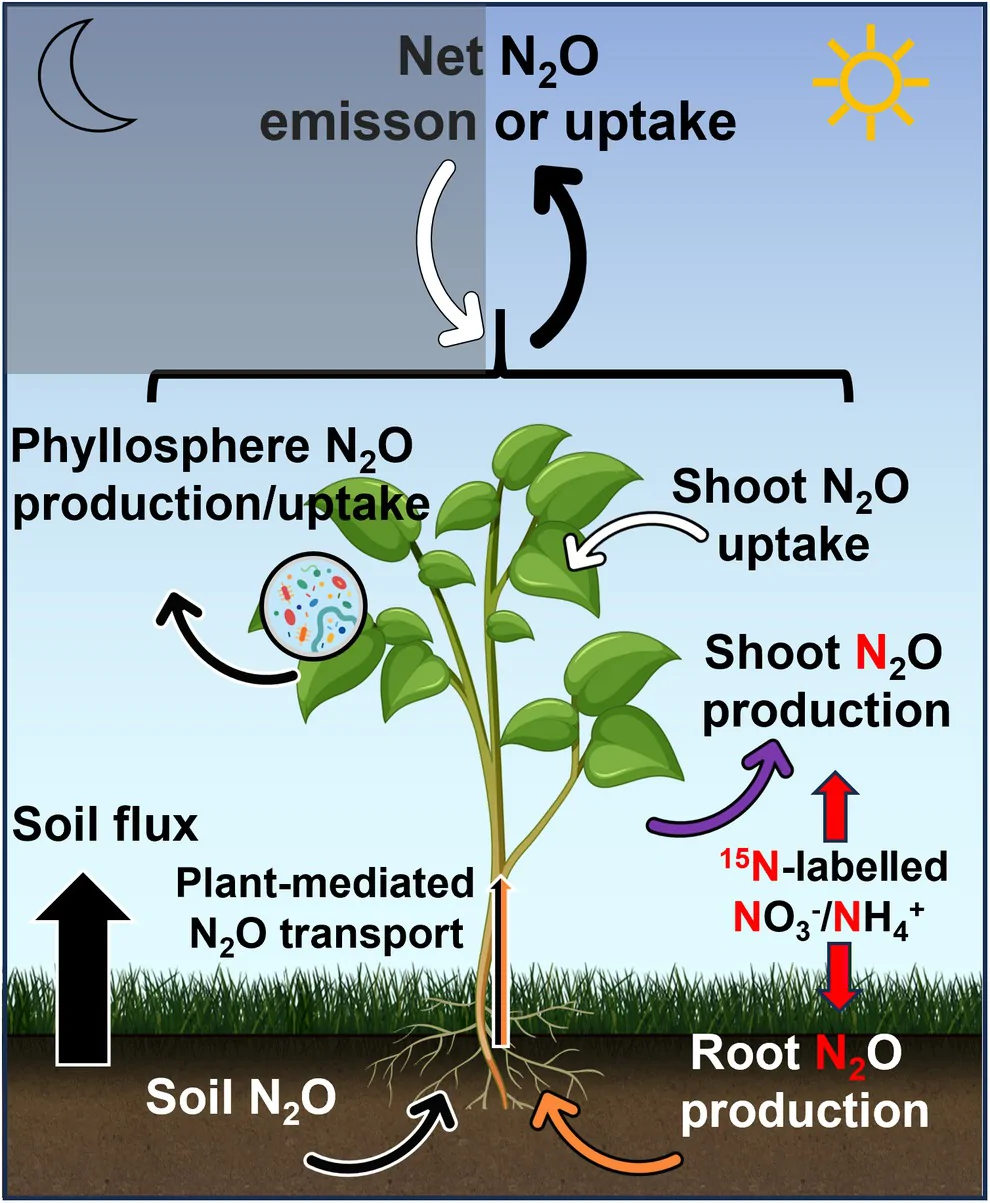
Conceptual scheme of the nitrous oxide (N_2_O) production, consumption, and transport processes across the soil–plant–atmosphere continuum. Processes established by prior literature are shown by black and white arrows; processes newly characterized at the organ level by this study, including the mechanistic distinction between root and shoot N_2_O formation pathways via isotope labelling (red arrows) and the light‐dependent source‐sink switch in shoots, are highlighted in orange and purple arrows.

Using sterile chicory cultures combining concentration measurement and ^15^N stable isotope labelling, we confirm that both roots and shoots independently produce and emit N_2_O without microbial influence. ^15^N enrichment in emitted N_2_O from root cultures supplied with ^15^N‐labelled NO_3_^−^, accompanied with negligible isotopic shifts in medium‐only controls, provides strong evidence that plant tissue rather than microbial activity was the dominant source of labelled N_2_O under these sterile conditions. All medium‐only controls showed negligible changes in δ^15^N–N_2_O values, confirming that plant tissue drove observed isotopic enrichment in emitted N_2_O, though a slight increase in δ^15^N–N_2_O in the ‘medium + ^15^NO_3_^−^ + dark’ control may reflect a minor abiotic contribution. However, given the low changes in N_2_O mixing ratios in these controls compared to the plant treatments, this is not considered to have meaningfully affected our results.

Most current models treat microbial activity as the primary N_2_O source and represent plant contributions as passive conduit transport of soil‐derived N_2_O. Our results show that N_2_O dynamics in the soil–plant‐atmosphere continuum are more complex: N_2_O produced in roots and shoots of distinct biochemical origin may mix as it moves upwards through the plant, with some fraction potentially reduced within plant cells. These organ‐specific processes must be incorporated into future vegetation N_2_O emission models, which currently do not account for them (Lenhart *et al*., 2019; Qin *et al*., 2024).

### Root N_2_O production: respiratory hypoxia and an NO_3_ ^−^‐dependent reductive pathway

Root cultures showed clear ^15^N enrichment in N_2_O when supplied with ^15^N–NO_3_^−^ but not ^15^N–NH_4_^+^, confirming that NO_3_^−^, but not NH_4_^+^, serves as the primary N_2_O precursor in roots, consistent with earlier work (Goshima *et al*., 1999; Lenhart *et al*., 2019). Crucially, the ‘root + ^15^N–NO_3_^−^’ treatment was the only treatment where substantial amounts of N_2_O were produced and in which both δ^15^N^α^–N_2_O and δ^15^N^β^–N_2_O increased in similar proportions (Fig. 5). This position‐specific enrichment was relatively symmetric; that is, it showed a smaller intramolecular difference than the ‘shoots + ^15^N–NO_3_^−^ + dark’ treatment. As one possible explanation, this pattern may reflect the isotopic fingerprint of N_2_O formation via nitric oxide as an intermediate: when two NO molecules, both originating from the same ^15^N labelled nitrate pool, combine to form N_2_O, ^15^N is distributed between the central (α) and terminal (β) nitrogen positions to a similar degree. We interpret this only as a relative comparison between organs and not as an assignment of a specific pathway, since the underlying δ^15^N^SP^–N_2_O value is a mass‐balance‐derived (the measured *δ* values remained near the calibrated range) and natural abundance site preference values (e.g. Sutka *et al*., 2006) might not apply in a ^15^N labelling experiment. The mass‐balance‐derived isotopic composition of produced N_2_O in the ‘root + ^15^NO_3_^−^’ treatment reflects this clearly, with δ^15^N^bulk^–N_2_O values of 635 ± 91‰, δ^15^N^α^–N_2_O values of 716 ± 102‰ and δ^15^N^β^–N_2_O values of 557 ± 80‰, indicating near‐proportional enrichment at both positions. By contrast, the ‘root +^15^NH_4_^+^’ treatment showed substantially lower source values across all metrics (δ^15^N^bulk^–N_2_O: 117 ± 6‰, δ^15^N^α^–N_2_O: 161 ± 16‰, δ^15^N^β^–N_2_O: 74 ± 6‰), consistent with limited incorporation of ammonium‐derived nitrogen into N_2_O via this pathway. The δ^15^N^SP^–N_2_O values further support a distinction between the two treatments. Total δ^15^N^SP^–N_2_O values were higher in the ‘root + ^15^NO_3_^−^’ treatment (160 ± 23‰) than in the ‘root + ^15^NH_4_^+^’ treatment (86 ± 20‰). The ‘root ^15^NH_4_^+^ treatment’ showed stronger relative enrichment at the α‐position compared to the β‐position. This suggests substrate‐dependent differences in ^15^N distribution within the plant and may be consistent with N_2_O formation via the phytoglobin–NO cycle and/or mitochondrial cytochrome c oxidase in nitrate‐supplied roots (Timilsina *et al*., 2020).

The probable mechanistic driver of this pathway is root respiratory hypoxia. Elevated CO_2_ production and O_2_ consumption in root incubations (Fig. 4b,c) indicate high respiratory demand that progressively depletes internal O_2_. Under these low‐oxygen conditions, reduction in NO_2_^−^ to NH_4_^+^ by nitrite reductase is inhibited, favouring NO accumulation and its subsequent conversion to N_2_O. The correlation between N_2_O production and respiration rates in our data is consistent with the central mitochondrial role proposed by Timilsina *et al*. (2020, 2022a).

### Shoot N_2_O production: oxygen‐sensitive pathway distinct from roots

The shoot data reveal a mechanistically informative divergence from expectations based on respiratory O_2_ depletion alone. Under dark conditions with NO_3_^−^/NH_4_^+^ supplementation, shoots produced *c*. 23–27 pmol g^−1^ h^−1^, roughly 35% more N_2_O per unit biomass than roots under equivalent conditions, despite respiratory CO_2_ production and O_2_ consumption rates less than half those of roots (Fig. 4a–c). If N_2_O formation were simply a by‐product of respiratory O_2_ depletion, roots should outproduce shoots on a per‐biomass basis. The fact that shoots exceed root N_2_O production under conditions of comparatively low respiratory activity suggests that shoot N_2_O formation operates through a pathway governed by mechanisms distinct from mitochondrial hypoxic NO reduction.

A different N_2_O formation pathway in shoots compared to roots is also supported by the isotopic data. Both root + ^15^NO_3_^−^ and shoot + ^15^NO_3_^−^+ dark treatments showed marked ^15^N enrichment in produced N_2_O, indicating that NO_3_^−^ serves as an N_2_O precursor in shoots as well as roots. Critically, however, the two treatments differed in the intramolecular distribution of ^15^N: roots supplemented with ^15^NO_3_^−^ showed relatively symmetric enrichment across the α‐ and β‐positions of N_2_O, whereas the shoot + ^15^NO_3_^−^ + dark treatment exhibited a markedly asymmetric pattern (mass‐balance‐derived δ^15^N^SP^–N_2_O values of 160 ± 23‰ and 429 ± 102‰, respectively; Fig. 5). We suggest that this asymmetry in site preference enrichment is indicative of a distinct N_2_O‐forming pathway in shoots compared to roots, while the dependence on NO_3_^−^ as substrate is retained in both organs.

By contrast, ^15^NH_4_^+^ was not detectably incorporated into N_2_O in shoots under dark conditions: the shoot + ^15^NH_4_^+^ + dark treatment (δ^15^N^bulk^–N_2_O: 66 ± 10‰, δ^15^N^α^–N_2_O: 119 ± 27‰, δ^15^N^β^–N_2_O: 14 ± 7‰, δ^15^N^SP^–N_2_O: 105 ± 33‰) was isotopically indistinguishable from the unlabelled shoot + dark control (δ^15^N^bulk^–N_2_O: 64 ± 24‰, δ^15^N^α^–N_2_O: 112 ± 28‰, δ^15^N^β^–N_2_O: 18 ± 21‰, δ^15^N^SP^–N_2_O: 94 ± 11‰), confirming that NH_4_^+^ does not serve as a significant N_2_O precursor in shoots. Because the shoot + dark‐only treatment received no ^15^N label, its δ^15^N values represent the natural isotopic baseline of shoot‐emitted N_2_O, reflecting fractionation during N_2_O formation from endogenous nitrogen pools rather than tracer incorporation. Medium‐only controls, including the medium + ^15^NH_4_^+^ control, showed negligible enrichment across all metrics, confirming that the observed isotopic signals originate from plant tissue activity rather than abiotic processes. Notably, the shoot + ^15^NH_4_^+^ + dark treatment remained at this isotopic baseline, indicating that the N_2_O emitted by shoots without ^15^NO_3_^−^ originates from endogenous, unlabelled nitrogen rather than from the supplied ammonium.

### Photosynthetic suppression of shoot N_2_O formation and the emergence of net uptake

Photosynthetic activity suppressed shoot N_2_O production by 88% in the ‘NO_3_^−^ + light’ treatment and induced net N_2_O uptake in the ‘NH_4_^+^ + light’ treatment. This differential response, with more complete suppression under NH_4_^+^ than NO_3_^−^, may reflect the distinct enzymatic pathways involved: NO_3_^−^‐derived N_2_O production proceeds via well‐characterized reductive steps (NO_3_^−^ → NO_2_^−^ → NO → N_2_O), whereas N_2_O formation from NH_4_^+^ requires prior oxidation to NO_2_^−^ or NO, a pathway less directly connected to the major nitrogen assimilation routes and therefore potentially more sensitive to disruption by photosynthetically elevated O_2_ and enhanced nitrite reductase activity.

The suppression mechanism operates through at least three linked steps. First, photosynthesis elevates internal O_2_, disfavouring cytochrome c oxidase‐mediated NO reduction. Second, light enhances nitrite reductase activity, efficiently converting NO_2_^−^ to NH_4_^+^ and thereby eliminating a key N_2_O precursor. Third, an as‐yet uncharacterized plant enzymatic activity may convert produced N_2_O to N_2_ possibly explaining net uptake rather than simply reduced production; canonical bacterial N_2_O reductase (NosZ) is O_2_‐sensitive and therefore unlikely to account for this effect under photosynthetic conditions, but the occurrence of N_2_ emissions from plants and net N_2_O consumption in the light suggest a distinct, possibly O_2_‐tolerant consumption pathway in plant tissue. This is consistent with reports of N_2_ emissions from wheat supplied with labelled NO_2_^−^ (Vanecko & Varner, 1955), with the negative correlation between N_2_O consumption and CO_2_ respiration across plant taxa (Timilsina *et al*., 2020), and with Schützenmeister *et al*. (2020), who found up to 63% reduction in N_2_O production during photosynthesis in ash and beech saplings. Net N_2_O uptake by European beech under certain conditions (Machacova *et al*., 2024) further supports the existence of an active plant N_2_O consumption pathway.

A ‘shoot + light’ control without ^15^N label could not be included due to instrument failure, which also prevented additional measurements from being conducted. Despite these limitations, the suppression of N_2_O formation under illumination is supported by the consistently low ^15^N enrichment across all light treatments irrespective of substrate, indicating that minimal net N_2_O flux carries little ^15^N label and that the light response does not depend on any single treatment.

### Implications for global N_2_O budgets: towards organ‐resolved, diel‐dynamic vegetation flux models

The findings presented here motivate concrete revisions to how plant‐mediated N_2_O is represented in global budgets, in two respects:

First, mechanistic resolution at the organ level. Currently, plant N_2_O production is conceptually represented as a single process. The results presented here require that representation to be split: roots operate a hypoxia‐associated, NO_3_^−^‐dependent reductive pathway that may involve NO as an intermediate; shoots operate through a distinct, oxygen‐sensitive mechanism with a light‐regulated source‐sink switch.

Second, the diel dynamic. The night‐time source and daytime sink behaviour of shoots, indicated by the dark and light treatments, creates a strongly time‐structured plant N_2_O flux. Vegetation models that apply a single emission factor regardless of time of day or photosynthetic status will both misattribute the timing and potentially misestimate the magnitude of plant contributions. Integrating the light‐switch mechanism identified here into land‐surface models represents a necessary next step, though further studies under natural conditions are needed to better characterize the magnitude and environmental controls of this process.

The plants used in this study were cultivated under sterile conditions, substantially reducing potential microbial contributions to N_2_O production, although their complete exclusion cannot be guaranteed. In addition, the HRCs investigated here are most representative of young fine roots rather than mature roots of soil‐grown plants. Future studies should therefore extend this isotope‐based approach to intact plants in soil mesocosms. This will enable simultaneous partitioning of root, shoot, and soil N_2_O fluxes and provide a framework for evaluating the magnitude and environmental controls of plant‐derived N_2_O production under more natural conditions. By comparing sterile and nonsterile systems, the position‐specific isotope labelling approach demonstrated here could further be used to discriminate N_2_O of plant‐metabolic origin from that produced by associated microorganisms such as endophytes (Qin *et al*., 2024). This would allow the proportion of plant‐associated N_2_O attributable to the plant itself to be constrained, which we consider an important direction for future research.

## Competing interests

None declared.

## Author contributions

MS was involved in conceptualization, methodology, investigation, formal analysis, data curation, visualization, writing – original draft, writing – review and editing, project administration. MM was involved in methodology, investigation, formal analysis, data curation, visualization, writing – original draft, writing – review and editing. TK was involved in conceptualization, writing – review and editing. SG was involved in conceptualization, methodology, resources, writing – review and editing, project administration, supervision, funding acquisition. FK was involved in conceptualization, methodology, resources, writing – review and editing, project administration, supervision, funding acquisition.

## Disclaimer

The New Phytologist Foundation remains neutral with regard to jurisdictional claims in maps and in any institutional affiliations.

## Supporting information


**Fig. S1** Flow chart summarizing the processing steps applied to convert the raw measurement results into the final corrected results.
**Fig. S2** Dependence of the measured ^15^N isotopic composition of nitrous oxide on the oxygen mixing ratio, used to derive the corresponding correction for δ^15^N^bulk^, δ^15^N^α^, and δ^15^N^β^.
**Fig. S3** Dependence of the measured ^15^N isotopic composition of nitrous oxide on the carbon dioxide mixing ratio, used to derive the corresponding correction for δ^15^N^bulk^, δ^15^N^α^, and δ^15^N^β^.
**Fig. S4** Dependence of the measured ^15^N isotopic composition of nitrous oxide on the methane mixing ratio, used to derive the corresponding correction for δ^15^N^bulk^, δ^15^N^α^, and δ^15^N^β^.
**Fig. S5** Dependence of the measured ^15^N isotopic composition of nitrous oxide (N_2_O) on the N_2_O mixing ratios below 330 parts per billion by volume, used to derive the corresponding correction for δ^15^N^bulk^, δ^15^N^α^, and δ^15^N^β^.
**Fig. S6** Change in the measured nitrous oxide mixing ratio over storage time for three septum types, used to derive the corresponding correction.
**Fig. S7** Change in the measured ^15^N isotopic composition of nitrous oxide (N_2_O) over storage time for white silicone/polytetrafluoroethylene septa, used to derive the corresponding correction for δ^15^N^bulk^, δ^15^N^α^, and δ^15^N^β^.
**Fig. S8** Change in the measured ^15^N isotopic composition of nitrous oxide over storage time for blue silicone/polytetrafluoroethylene septa, used to derive the corresponding correction for δ^15^N^bulk^, δ^15^N^α^, and δ^15^N^β^.
**Fig. S9** Change in the measured ^15^N isotopic composition of nitrous oxide over storage time for butyl/polytetrafluoroethylene septa, used to derive the corresponding correction for δ^15^N^bulk^, δ^15^N^α^, and δ^15^N^β^.
**Fig. S10** Matrix of *P*‐values for all pairwise treatment comparisons across the measured gas exchange rates and the nitrous oxide isotope source parameters.
**Method S1** Underlying principles of cross‐sensitivities during analysis of nitrous oxide mixing ratios and isotope values using cavity ring‐down spectroscopy.
**Method S2** Underlying principles of the effect of vials, septa and storage time on cavity ring‐down based nitrous oxide analysis.
**Method S3** Processing and correction of raw data.
Please note: Wiley is not responsible for the content or functionality of any Supporting Information supplied by the authors. Any queries (other than missing material) should be directed to the *New Phytologist* Central Office.

## Data Availability

The data supporting the findings of this study have been deposited in heiDATA, the institutional research data repository of Heidelberg University (doi: 10.11588/DATA/Y8B6MU, Schroll *et al*., 2026).
