# Supplementary material for: Insights into plant‐part‐specific N2O production in roots and shoots of chicory (Cichorium intybus) using stable isotope labelling
Source: New Phytol. 2026 Sep 15;252(4):1432–44. doi: 10.1111/nph.71565 (PMC13669802; doi:10.1111/nph.71565)
Supplement: Supplementary file 1 [file NPH-252-1432-s001.docx]

## *New Phytologist* Supporting Information

Article title: Insights into plant-part specific N_2_O production in roots and shoots of chicory (*C. intybus*) using stable isotope labelling

Authors: Moritz Schroll, Maurice Maas, Steffen Greiner, Thomas Klintzsch, Frank Keppler

Article acceptance date: 19 August 2026

The following Supporting Information is available for this article:

**Method S1** Underlying principles of cross-sensitivities during analysis of N_2_O mixing ratios and isotope values using cavity ring-down spectroscopy.

CRDS instruments, like the Picarro G5131-*i* used in this study, are generally susceptible to be affected by cross-sensitivities. This term refers to the influence of other gases (e.g. O_2_, CO_2_ or CH_4_) on the analytical result of the target gas (e.g., Harris *et al.*, 2020), in this case, N_2_O. Additionally to cross-sensitivities, the mixing ratio of the target gas itself may have potential impact on analytical results, too. To account for these analytical problems, correction factors need to be applied to the results obtained from analytical measurements. These factors are determined through experiments involving known mixing ratios of the respective gases and their impact on measurements of N_2_O mixing ratio and isotopic composition. For the present study, these corrections were specifically established for the used Picarro G5131-*i* device. While previous studies have derived correction factors for common gases affecting this type of instrument, the specific effects of cross-sensitivities are device-dependent, necessitating the determination of correction factors through independent experiments (Harris *et al.*, 2020). In this study, we derived correction factors using linear regression analysis for the influence of O_2_, CO_2_, and CH_4_, as well as for different N_2_O mixing ratios, on the measurements obtained with the Picarro G5131-*i*. Consistent with prior research, we observed a statistically significant correlation between the mixing ratios of O_2_, CO_2_, and CH_4_ and the measured N_2_O mixing ratio and isotopic values. The exact methodology and sequence of applying these correction factors to the raw data are detailed in Method S3.

**Method S2** Underlying principles of the effect of vials, septa and storage time on cavity ring-down based N_2_O analysis.

The selection of vials and septa represents, together with cross-sensitivities, a major source of analytical error in CRDS-based N_2_O analysis. In some samples, we detected N_2_O mixing ratios below atmospheric levels, which cannot be explained with N_2_O loss related to diffusion. This effect was predominantly observed in control group samples, thereby excluding biological N_2_O degradation as a potential cause. These findings suggest that interactions between air samples and the materials of the vials or the white silicone-PTFE septa (IVA-Analysentechnik / manufacturer) have the potential to influence the analytical results. Underlying mechanisms could be physical and chemical processes such as absorption, desorption, or chemical reactions between N_2_O and the material of vials and septa (Laughlin & Stevens, 2003; Rochette & Bertrand, 2003). The logical choice for storage vials would have been Exetainers (Labco Limited, UK), as they were also used for gas chromatographic analysis of O_2_, CO_2_ and CH_4_ during this study and show the best results in relation to storage, tightness and N_2_O loss (Glatzel & Well, 2007). They also showed good results in tests regarding *δ*^15^N-N_2_O measurements via IRMS (Laughlin & Stevens, 2003). However, in our own experiments Exetainers revealed a significant shift in *δ*^15^N-values even shortly after gas injecting into Exetainers, which renders them unsuitable for our specific analytical requirements. Possible reason could likely be outgassing of septa compounds interfering with CRDS measurements. To address this issue, we conducted experiments evaluating three different septa types: blue silicone-PTFE septa (Chromatographie-Zubehör Trott / manufactuer), white silicone-PTFE septa (IVA-Analysentechnik / manufacturer) and butyl-PTFE septa (IVA-Analysentechnik / manufacturer). These experiments involved filling vials with ambient air and monitoring the changes in measured N_2_O mixing ratios and N_2_O isotopic composition over time. The results indicate that all three of the used septa types affected N_2_O mixing ratios and *δ*^15^N-N_2_O values. This effect was the smallest for the butyl-PTFE septa (IVA-Analysentechnik / manufacturer), making them the choice for subsequent experiments. Still, correction factors were required to account for these effects, which got stronger with increasing storage time. Thus, we derived time-dependent correction factors via linear regression analysis for the white silicone-PTFE septa (IVA-Analysentechnik / manufacturer) used in the earlier experiments and for the butyl-PTFE septa (IVA-Analysentechnik / manufacturer) we used in later experiments of this study. These correction factors have a temporal component to account for the different storage time of samples. The exact methodology and sequence of applying these correction factors to the raw data are detailed in Fig. S1 and Method S3.

**Fig. S1** Flow chart summarizing the processing steps applied to convert the raw measurement results into the final corrected results. Boxes denote individual processing or correction steps and arrows indicate the order in which they are applied.

**Method S3** Processing and correction of raw data.

As outlined above, cross-sensitivities with other gases and low N_2_O mixing ratios, as well as septa, vials, and storage, influence the measurements of N_2_O mixing ratios and isotopic composition using the Picarro G5131-*i*. To minimize these effects, corrections were applied to the measurement results. Fig. S1 illustrates the workflow and the sequential steps of the corrections as applied during this study. The subsequent chapters provide a more detailed explanation of each correction step. The largest corrections applied were 30,7 ppb for the N_2_O mixing ratio, 20.5 ‰ for *δ*^15^N^bulk^, 37.3 ‰ for ^15^N^α^ and 10.9 ‰ for ^15^N^β^ values.

**Analysis of CH_4_ mixing ratios**

CH_4_ mixing ratios were determined using the same method as for CO_2_ and O_2_ mixing ratios (see Material and Methods), with a GC-2010 Plus gas chromatograph (Shimadzu, Japan) coupled with a barrier discharge ionization detector (BID-2010 Plus, Shimadzu, Japan) being used. (Setup: packed stainless-steel column (length: 2 m, inner diameter: 0.53 mm), stationary phase: carbon molecular sieve (ShinCarbon ST, 80/100 Mesh; Supelco, USA), carrier gas: High-purity helium (grade 6.0, Air Liquide, France) at a constant flow rate of 5 mL min^-1^).

The sample injection was performed using an autosampler (AOC-20i, Shimadzu, Japan). As part of quality control, one reference standard was measured after every six to nine single measurements. Calibration was performed with certified standard gas mixtures (CRYSTAL Gas Mixture, Air Liquide, France). Methane quantification was based on analyses of multiple reference standards (CH_4_: 50–25,000 ppmv), measured in triplicates.

**Correction for cross-sensitivities**

The raw isotopic data were corrected for cross-sensitivities of O_2_, CO_2_, and CH_4_. As an example, the correction of the *δ*^15^N^bulk^ value for the cross-sensitivity with O_2_ is illustrated using the following equations (Eqs. 1, 2 and 3). First, the difference (ΔO_2_) between the O_2_ mixing ratio of the sample and that of the standard gas was determined:

$\text{∆}\text{O}_{\text{2}} = \text{O}_{\text{2}}\text{ }\left( \text{sample} \right)-\text{O}_{\text{2}}\text{ }\left( \text{standard gas} \right)$

(1)

Subsequently, the change in *δ*^15^N^bulk^ (Δ*δ*^15^N^bulk^) due to the cross-sensitivity with O_2_ was calculated using the determined slope (m) of the regression line from experimental trials and ΔO_2_ determined from equation 1:

$$\text{∆}\text{δ}^{\text{15}}\text{N}^{\text{bulk}}\text{ }=\text{ }\text{m }\times\text{ ∆}\text{O}_{\text{2}}$$

(2)

The final corrected *δ*^15^N^bulk^ value (denoted as *δ*^15^N^bulk^ (C)) was obtained by adding Δ*δ*^15^N^bulk^ from the previous equation to the initial measured value (*δ*^15^N^bulk^):

$$\text{δ}^{\text{15}}\text{N}^{\text{bulk}}\text{ (C)}=\text{ }\text{∆}\text{δ}^{\text{15}}\text{N}^{\text{bulk}}+\text{δ}^{\text{15}}\text{N}^{\text{bulk}}$$

(3)

Additionally to the correction for cross-sensitivities all measurement results with a N_2_O mixing ratio below 330 ppbv were corrected for the influence of a low N_2_O mixing ratio on isotopic measurements of N_2_O. As an example, the correction of the *δ*^15^N^bulk^ value is showed in the equations below (Eqs. 4 and 5). The difference in *δ*^15^N^bulk^ (Δ*δ*^15^N^bulk^) caused by the low N_2_O mixing ratio was determined using the difference between 330 and the N_2_O mixing ratio and the slope (m) of the regression line from experimental trials (Figs S1-4):

$$\text{∆}\text{δ}^{\text{15}}\text{N}^{\text{bulk}}\text{ }=\text{ }\text{m }\times\text{ (330 }-\text{ N}_{\text{2}}\text{O [}\text{ppbv}\text{])}$$

(4)

**Fig. S2** Dependence of the measured ^15^N isotopic composition of nitrous oxide (N_2_O) on the oxygen (O_2_) mixing ratio, used to derive the corresponding correction for δ^15^N^bulk^ (green), δ^15^N^α^ (blue), δ^15^N^β^ (yellow). Dotted lines are linear regression fits from which the correction factors were derived, with the coefficient of determination (R^2^) given for each fit.

**Fig. S3** Dependence of the measured ^15^N isotopic composition of nitrous oxide (N_2_O) on the carbon dioxide (CO_2_) mixing ratio, used to derive the corresponding correction for δ^15^N^bulk^ (green), δ^15^N^α^ (blue), δ^15^N^β^ (yellow). Dotted lines are linear regression fits from which the correction factors were derived, with the coefficient of determination (R^2^) given for each fit.

**Fig. S4** Dependence of the measured ^15^N isotopic composition of nitrous oxide (N_2_O) on the methane (CH_4_) mixing ratio, used to derive the corresponding correction for δ^15^N^bulk^ (green), δ^15^N^α^ (blue), δ^15^N^β^ (yellow). Dotted lines are linear regression fits from which the correction factors were derived, with the coefficient of determination (R^2^) given for each fit.

**Fig. S5** Dependence of the measured ^15^N isotopic composition of nitrous oxide (N_2_O) on the N_2_O mixing ratios below 330 parts per billion by volume (ppbv), used to derive the corresponding correction for δ^15^N^bulk^ (green), δ^15^N^α^ (blue), δ^15^N^β^ (yellow). Dotted lines are linear regression fits from which the correction factors were derived, with the coefficient of determination (R^2^) given for each fit.

The calculated value (Δ*δ*^15^N^bulk^) was then added to the initial measured value (*δ*^15^N^bulk^) to determine the corrected *δ*^15^N^bulk^ value (*δ*^15^N^bulk^ (C):

$$\text{δ}^{\text{15}}\text{N}^{\text{bulk}}\text{ (C)}=\text{ }\text{∆}\text{δ}^{\text{15}}\text{N}^{\text{bulk}}+\text{δ}^{\text{15}}\text{N}^{\text{bulk}}$$

(5)

**Correction for influence of septa, vials and storage**

In addition to the correction of the influence of cross-sensitivities from other gases and for the influence of N_2_O itself, the measurement results need to be corrected for the influence of septa and vials used for sample storage. The longer the period between sampling and measurement, the greater the effect of the septa on the measured values. The *δ*^15^N^bulk^ value is used as an example for the correction (Eq. 6):

| $\text{δ}^{\text{15}}\text{N}^{\text{bulk}}\text{ (C) }=\text{ }\text{δ}^{\text{15}}\text{N}^{\text{bulk}} \times\text{ m }\times\text{ t}$ [h] | (6) |
| --- | --- |

*δ*^15^N^bulk^ refers to the initial measurement value, m is the slope of the regression line determined from the experimental trials, t is the time during which the sample remained in the vial with respective septa, and *δ*^15^N^bulk^ (C) is the corrected value. Corrections factors of the respective septa can be found in Figs. S5-9:

**Fig. S6** Change in the measured nitrous oxide (N_2_O) mixing ratio over storage time for three septum types, used to derive the corresponding correction. Data are shown for white silicone/PTFE (polytetrafluoroethylene; green), blue silicone/PTFE (yellow) and butyl/PTFE septa (brown). Dotted lines are linear regression fits from which the correction factors were derived, with the coefficient of determination (R^2^) given for each fit.

**Fig. S7** Change in the measured ^15^N isotopic composition of nitrous oxide (N_2_O) over storage time for white silicone/PTFE (polytetrafluoroethylene) septa, used to derive the corresponding correction for δ^15^N^bulk^ (green), δ^15^N^α^ (blue), δ^15^N^β^ (yellow). Dotted lines are linear regression fits from which the correction factors were derived, with the coefficient of determination (R^2^) given for each fit.

**Fig. S8** Change in the measured ^15^N isotopic composition of nitrous oxide (N_2_O) over storage time for blue silicone/PTFE (polytetrafluoroethylene) septa, used to derive the corresponding correction for δ^15^N^bulk^ (green), δ^15^N^α^ (blue), δ^15^N^β^ (yellow). Dotted lines are linear regression fits from which the correction factors were derived, with the coefficient of determination (R^2^) given for each fit.

**Fig. S9** Change in the measured ^15^N isotopic composition of nitrous oxide (N_2_O) over storage time for butyl/PTFE (polytetrafluoroethylene) septa, used to derive the corresponding correction for δ^15^N^bulk^ (green), δ^15^N^α^ (blue), δ^15^N^β^ (yellow). Dotted lines are linear regression fits from which the correction factors were derived, with the coefficient of determination (R^2^) given for each fit.

**Correction of potential instrument drift**

One in-house reference gas for N_2_O mixing ratios and one reference standard gas (RM 2; Mohn *et al.*, 2022) were analyzed at the beginning and end of each measurement day, allowing correction of potential instrumental drift by applying a linear two-point normalization following Paul *et al.* (2007).

**Fig. S10** Matrix of p-values for all pairwise treatment comparisons across the measured gas exchange rates and the N_2_O isotope source parameters. Panels show the comparisons for (a) the N_2_O production rate, (b) the CO_2_ production rate, (c) the O_2_ uptake rate, (d) the bulk δ^15^N value of N_2_O (δ^15^N^bulk^), (e) the δ^15^N value at the central α position (δ^15^N^α^), (f) the δ^15^N value at the terminal β position (δ^15^N^β^) and (g) the δ^15^N site preference (δ^15^N^SP^). Each cell shows the p-value from a pairwise t-test. Asterisks denote significance levels (* p < 0.05, ** p < 0.01, *** p < 0.001) and shaded cells denote statistically significant differences.

**References**

Glatzel S, Well R. 2007. Evaluation of septum-capped vials for storage of gas samples during air transport. *Environmental Monitoring and Assessment* 136: 307–311.

Harris SJ, Liisberg J, Xia L, Wei J, Zeyer K, Yu L, Barthel M, Wolf B, Kelly BFJ, Cendón DI, *et al.* 2020. N_2_O isotopocule measurements using laser spectroscopy: analyzer characterization and intercomparison. *Atmospheric Measurement Techniques* 13: 2797–2831.

Laughlin RJ, Stevens RJ. 2003. Changes in Composition of Nitrogen‐15‐Labeled Gases during Storage in Septum‐Capped Vials. *Soil Science Society of America Journal* 67: 540–543.

Mohn J, Biasi C, Bodé S, Boeckx P, Brewer PJ, Eggleston S, Geilmann H, Guillevic M, Kaiser J, Kantnerová K, *et al.* 2022. Isotopically characterised N_2_O reference materials for use as community standards. *Rapid Communications in Mass Spectrometry* 36: e9296.

Paul D, Skrzypek G, Fórizs I. 2007. Normalization of measured stable isotopic compositions to isotope reference scales – a review. *Rapid Communications in Mass Spectrometry* 21: 3006–3014.

Rochette P, Bertrand N. 2003. Soil air sample storage and handling using polypropylene syringes and glass vials. *Canadian Journal of Soil Science* 83: 631–637.
